# Reliability and relative validity of a child nutrition questionnaire to simultaneously assess dietary patterns associated with positive energy balance and food behaviours, attitudes, knowledge and environments associated with healthy eating

**DOI:** 10.1186/1479-5868-5-5

**Published:** 2008-01-29

**Authors:** Annabelle M Wilson, Anthea M Magarey, Nadia Mastersson

**Affiliations:** 1 Department of Nutrition and Dietetics, Flinders University of South Australia, Bedford Park, South Australia; 2 Southern Primary Health Service, Southern Primary Health, Noarlunga Centre, South Australia

## Abstract

**Background:**

Food behaviours, attitudes, environments and knowledge are relevant to professionals in childhood obesity prevention, as are dietary patterns which promote positive energy balance. There is a lack of valid and reliable tools to measure these parameters. The aim of this study was to determine the reliability and relative validity of a child nutrition questionnaire assessing all of these parameters, used in the evaluation of a community-based childhood obesity prevention project.

**Methods:**

The development of the 14-item questionnaire was informed by the aims of the obesity prevention project. A sub-sample of children aged 10–12 years from primary schools involved in the intervention was recruited at the project's baseline data collection (Test 1). Questionnaires were readministered (Test 2) following which students completed a 7-day food diary designed to reflect the questionnaire. Twelve scores were derived to assess consumption of fruit, vegetables, water, noncore foods and sweetened beverages plus food knowledge, behaviours, attitudes and environments. Reliability was assessed using (a) the intra class correlation coefficient (ICC) and 95% confidence intervals to compare scores from Tests 1 and 2 (test-retest reliability) and (b) Cronbach's alpha (internal consistency). Validity was assessed with Spearman correlations, bias and limits of agreement between scores from Test 1 and the 7-day diaries. The Wilcoxon signed rank test checked for significant differences between mean scores.

**Results:**

One hundred and forty one students consented to the study. Test 2 (n = 134) occurred between eight and 36 days after Test 1. For 10/12 scores ICCs ranged from 0.47–0.66 (p < 0.001) while for two scores ICCs were < 0.4 (p < 0.05). Spearman correlations ranged from 0.34–0.48 (p < 0.01) and Cronbach's alpha 0.50–0.80. Three scores were modified based on this analysis. The Wilcoxon signed rank test found no evidence of a difference between means (p > 0.05) for 10/12 (test-retest reliability) and 3/7 (validity) scores.

**Conclusion:**

This child nutrition questionnaire is a valid and reliable tool to simultaneously assess dietary patterns associated with positive energy balance, and food behaviours, attitudes and environments in Australian school children aged 10–12 years. Thus it can be used to monitor secular changes in these parameters and measure the effectiveness of this and other obesity prevention projects with similar aims.

## Background

There is a high and increasing prevalence of overweight and obesity worldwide [1-3]. The International Obesity Taskforce (IOTF) estimate the global prevalence of overweight and obesity in school-aged children to be 10% [2]. The co-morbidities of excess weight and the impact on the healthcare system indicate that obesity is a significant public health issue [4]. Hence, public health interventions are warranted to combat this increasing epidemic.

While there is a body of literature about managing childhood obesity, most interventions or programs are single setting and predominantly school based [5]. Furthermore, the evidence base for interventions aiming to prevent childhood obesity, particularly in community settings, is limited [5,6]. Recent international and country-specific reports highlight key principles likely to improve population weight status [6-9]. These include focusing on prevention during childhood, environmental change, working in partnership with a range of settings and sectors, using a portfolio and sufficient dose of interventions, and utilising a community development approach. Interventions utilising these approaches are also more likely to result in sustainable and equitable changes to health behaviours through successful environmental and community-based change.

**Figure 1:**
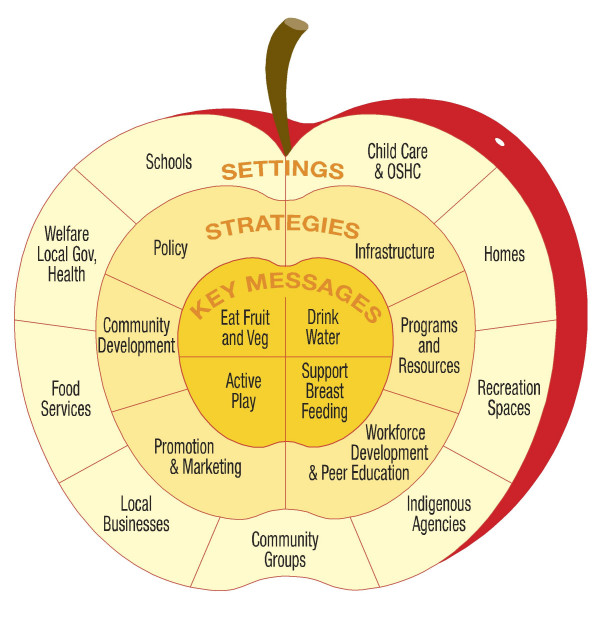
Key messages, settings and strategies of the *eat well be active *Community Programs.

The setting for the present study was the *eat well be active (ewba) *Community Programs, a community-based childhood obesity intervention project in South Australia implementing the key principles above. It addresses environmental and individual barriers to behaviour change in a range of settings (Figure 1) and aims to promote healthy weight in children aged 0–18 years through increasing healthy eating and physical activity behaviours [10]. The project is implemented through a range of strategies (Figure 1).

Comprehensive evaluation of this obesity prevention project and similar interventions is crucial in order to contribute to the evidence for effectiveness of childhood obesity interventions [11]. However, such evaluation is limited by the lack of setting specific tools (which ensure evaluation appropriate to the target group) and tools that evaluate the outcome, process and impact of these interventions. There are a relatively large number of valid and reliable tools that measure nutrient intake in school aged children [12-16]. However, there is a lack of tools that measure both (a) specific dietary patterns that increase the risk of positive energy balance and hence overweight and obesity, and (b) behaviours, attitudes, environments and knowledge associated with healthy eating.

While information regarding dietary patterns can be obtained from tools measuring nutrient intake, analyses are cumbersome, time consuming and costly [17], particularly when measuring large subject numbers as required in population-based projects. Additionally, the subject burden of the tool itself is usually high and there are methodological concerns in their use in school-aged children [12]. The lack of tools that encompassed the range of factors of interest in the *ewba *project required development of a project specific questionnaire. In order to be certain of the value of this new tool in assessing the effectiveness of the *ewba *programs it was necessary to determine its psychometric properties.

Thus the aim of this study was to determine the reliability and relative validity of a child nutrition questionnaire that simultaneously measures dietary patterns that promote the risk of positive energy balance, and food behaviours, attitudes, environments and knowledge in a sample of approximately 100 school children aged 10–12 years participating in a community-based obesity prevention project.

## Methods

Ethical approval for this study was obtained from the Flinders University Social and Behavioural Research and the Department of Education and Children's Services Ethics Committees.

### Subjects

The target sample size was 100 as this is recognised to give good precision for reliability and validity studies [18]. A convenience sample of primary school children in grades five to seven (approximate age 10–12 years) participating in baseline measures for the obesity prevention project was recruited for the present study. These students attended seven of the 44 primary schools that had been invited to and had agreed to participate in the obesity prevention project. These seven schools were booked into the early phase of baseline data collection for the obesity prevention project. Those students who had parental consent and child assent for the baseline measures were approached to be part of the present study. A mix of public and private, and metropolitan and rural primary schools was ensured.

### Development of the child nutrition questionnaire

The child nutrition questionnaire is one of seven program specific questionnaires developed within the obesity prevention project to ensure thorough evaluation of process and outcome elements. It was developed to measure changes specific to the key nutritional aims of the project (Figure 1) and was based on similar tools used in other parts of Australia (personal communication, Magarey AM & Dollman J). These aims arose from known dietary patterns which increase the risk of positive energy balance, notably increased consumption of noncore foods and sweetened beverages, and decreased consumption of fruit, vegetables and water [19,20]. These risk behaviours informed the development of the Action Plans which are the frameworks by which the obesity prevention project is implemented [10].

A key distinction of this questionnaire from others is that rather than a complete dietary assessment, it provides information on both (a) dietary patterns of interest to childhood obesity researchers and (b) behaviours, attitudes environments and knowledge associated with healthy eating. There are 14 questions with a variable number of items. A range of response options are used including five point Likert scales and a choice of frequencies relating to either usual or recent (previous/current day) intake (Table 1). It takes 10–12 year old children approximately 20 minutes to complete and provides categorical data.

**Table 1:** The 12 scores grouped into five categories, assessing intake, healthy behaviours, attitude, environment and knowledge, the individual items contributing to each score and the response for each item.

**Category ***Score (total items)*	Items in each score	No. of items	Response
**Intake**			

*Non-core food (14)*	*Consumption at recess, lunch or after school of following listed foods *potato crisps, chocolate, lollies, muesli bar, savoury biscuits, sweet biscuits, ice-cream/ice block, hot chips, pie/pasty/sausage roll, hot dog, pizza	11	Tick if consumed ^Ω^
	*No. times/week the following listed foods eaten: *chocolate/lollies, potato crisps, hot chips	3	Frequency scale A^ψ^
*Sweetened beverages *(6)	*Consumption at recess, lunch or after school of following listed drinks *Cordial, fruit juice, soft drink, diet soft drink	4	Tick if consumed^Ω^
	*No. times/week the following listed drinks consumed: *fruit juice, regular soft drink	2	Frequency scale A^ψ^
*Sweetened beverages without diet soft drink*	As above but without diet soft drink	5	
*Water (2)*	*Consumption of *water at recess, lunch or after school	1	Tick if consumed^Ω^
	no. times/week drink water	1	Frequency scale A^ψ^
*Fruit (4)*	*Consumption of *fresh and dried fruit consumed at recess, lunch or after school	2	Tick if consumed^Ω^
	Estimated number of fruit serves consumed/day	1	Frequency scale B^Σ^
	Number of different fruit consumed yesterday (from a list of 15)	1	Tick if consumed: reduced to none = 0, 1 = 1, 2–3 = 2, 4–5 = 3, 6–15 = 4
*Vegetables (3)*	Vegetables consumed at recess, lunch and after school	1	Tick if consumed^Ω^
	Estimated number of vegetable serves consumed/day	1	Frequency scale B^Σ^
	Number of different vegetables consumed yesterday (from a list of 25)	1	Tick if consumed: reduced to none = 0, 1–3 = 1, 4–6 = 2, 7–9 = 3, 10–25 = 4

**Healthy behaviour**			

*Healthy behaviour (8)*	*Number of times per week: *eat breakfast, carry water bottle, help with groceries, help prepare dinner, eat dinner with family, eat dinner in front of TV^§^, eat snacks in front of TV^§^, eat fast food^§^	8	Frequency scale A^ψ^

**Attitude**			

*Fruit (5)*	*With regards to fruit, agreement with: *makes me feel healthy^§^, tastes good^§^, easy snack^§^, I like tasting new fruits^§^, cheap^§^	5	Likert scale (1 to 5)*
*Vegetable (4)*	*With regards to vegetables, agreement with: *makes me feel healthy^§^, tastes good^§^, I like tasting new vegetables^§^, easy to prepare^§^	4	Likert scale (1 to 5)*

**Environment**			

*Fruit & vegetable (6)*	*With respect to fruit & vegetables, agreement with: *veg usually served at dinner^§^, fruit available to eat at home^§^, parents & teachers encourage fruit & veg consumption^§^	4	Likert scale (1 to 5)*
	Frequency of fruit & veg break at school	1	Frequency scale C^Φ^
	Number of fruits and vegetables (15 fruit, 25 veg) never consumed/don't know what they are	1	Tick if never consumed or don't know what they are: fruit recoded: none = 2, 1–3 = 1, 4–15 = 0; vegetable recoded: none = 3, 1–2 = 2, 3–6 = 1, 7–25 = 0; two values summed

**Knowledge**			

Fruit (1)	No. serves fruit should be consumed by a child of your age each day	1	Select from: none, < 1 a day, 1–2 a day, more than 5 a day
Vegetables (1)	No. serves vegetables should be consumed by a child of your age each day	1	Select from: none, < 1 a day, 1–2 a day, more than 5 a day

Ω *^Score^* 1 for each item consumed at each time point

ψ Frequency scale A: never/rarely, less than once/week, about 1–3 times per week, about 4–6 times per week, every day, given values 1–5 respectively

Σ Frequency scale B: I don't eat fruit (vegetable), less than 1 serve per day, 1–2 serves per day, 3–5 serves per day, more than 5 serves per day, given values 1–5 respectively

^§^Item reverse scored before summing

* Likert scale: strongly agree, agree, not sure, disagree, strongly disagree, given values 1–5 respectively

Φ Frequency scale C: never/rarely, once a week, most days a week, every day, given values 1–4 respectively

### Scoring of the child nutrition questionnaire

Twelve scores were developed from the questionnaire and placed into five categories (Table 1). A score includes several elements of a single construct. The rationale for using scores is that a score is more sensitive to change; hence when used for analysis of the effectiveness of the intervention, small changes are more likely to be identified. Scores were created by summing items specific to each message (fruit, vegetables, water, noncore foods or sweetened beverages) and parameter (intake, attitude, behaviour, knowledge or environment) (Table 1). Relevant items were reverse scored before summing (Table 1). To ensure consistency among all intake questions, responses measuring weekly intake were divided by seven to represent daily intake. In the intake category, the values for the number of different fruits (vegetables) consumed yesterday were reduced to a value between zero and four, and in the environment category the number of fruits and vegetables never consumed or not known was reduced to a number between zero and two and zero and three respectively (Table 1). The cut-points were based on the distribution within the sample and to be sensitive to change in intake. If one or more items within a score were missing that score was not calculated resulting in up to 20 less cases for some score comparisons.

### Piloting of the child nutrition questionnaire

The questionnaire was piloted in a convenience sample of seven grade five to seven students. These students attended primary schools not involved in the community-based project. After completing the questionnaire, students were briefly interviewed by the first author (AW) on its content and the proposed data collection process. Review of the completed surveys and child comments led to minor changes to the tool to improve comprehension and ability to respond. For example students had difficulty understanding how to answer questions four and five. Consequently these were repositioned to be questions one and two so that all students could be guided through them in a standardised way by a classroom helper.

### Reliability

#### Test 1 (Baseline measures)

The first administration of the questionnaire was a component of the baseline measures for the evaluation of the community-based obesity prevention project.

Standard administration of the questionnaire was followed at each school including an instruction preamble, poster depicting fruit and vegetable serve sizes and availability of two to three classroom helpers to assist with any student queries. Concurrently, students with parental consent and child assent to baseline anthropometry measures had height and weight measures collected out of view of other students, by trained team members. Height was measured using a stadiometer (Wedderburn, Model Number PE087, Australia and Germany) to the nearest 0.1 centimetre. Weight was taken using calibrated digital scales (Tanita, Model Number HD332, China) to the nearest 0.1 kilogram. Two measures were taken; a third only if the difference between the first two were too great (height: defined as > 5 mm difference). Mean height and weight were calculated, or the median was taken if three measures were required.

#### Test 2

On the day of Test 1, school principals were approached and given a letter outlining the process of questionnaire validation. After the school's agreement was obtained, consent forms and information regarding this study was handed out to all students who consented to and completed the nutrition questionnaire at baseline measures. Students who returned consent forms with parental consent and child assent by the time the questionnaires were readministered (Test 2) were included in this study. Schools were booked in for Test 2 on days and times mutually convenient for the school and first author (AW), not including Mondays. The questionnaire asks about intake the previous day and food intake had been shown to vary from weekdays to weekends [21] and thus Mondays were avoided to minimise this bias.

At Test 2, the questionnaire was administered as for Test 1. However, due to logistics, only one classroom helper was present. Height and weight measurements were not repeated.

### Relative validity

#### The *ewba *7-day diaries

Attitude and knowledge questions and some of the items in the environment category are unable to be validated in the traditional sense hence were not included in the evaluation of relative validity. Relative validation focussed on assessing whether what was reported to have been consumed (on the day of survey or in the last week) was reflective of usual behaviour. Seven (one for each day of the week) eight-page diaries were designed specifically to reflect the food intake content of the questionnaire. For each day, the individual foods (e.g. those to be ticked if consumed at recess, lunch or after school, those for which frequency in the last week was sought, the 15 fruits and 25 vegetables) and the behaviours (e.g. help prepare dinner, eat snacks in front of TV) were listed and respondents were requested to tick each time they consumed one of the listed items or partook in a behaviour. The two weekend days were slightly different from the weekdays, for example 'recess' was referred to as the 'morning snack' and 'after school' as the 'afternoon snack'. The diaries were developed by the first author (AW) in conjunction with the second author (AM) and aimed to determine frequency of intake, not quantity of relevant foods.

The 7-day diaries were distributed with a standard instruction sheet at Test 2, after completion of the questionnaire. Students were asked to begin completing them the day after Test 2 and return to the first author (AW) in the reply paid envelope as soon as completed. As an incentive to return diaries, the first two students who returned them from each school were sent a pen or magnet. If students had consented to the present study, completed Test 1 but were absent, the diaries were left with their classroom teacher to be distributed. Two weeks after Test 2, students were reminded by the school to return their diaries. Parents of students who had still not returned their diaries were phoned by the first author (AW) three weeks after Test 2.

For each subject information from the seven diaries was collated to determine a daily or weekly (as appropriate) value for each item in the questionnaire. These calculated values were summed to provide values for the 7 scores.

#### Use of 1995 National Nutrition Survey (NNS) database

In order to validate estimates of the number of serves of fruit and vegetables consumed, the daily fruit and vegetable frequency data from the diaries were converted to quantities based on the average weight of each of the listed 20 fruits and 30 vegetables as consumed by nine to 13-year old participants (n = 891) in the National Nutrition Survey 1995 [22,23]. Average daily fruit and vegetable serves consumed were calculated as total weight of each of fruit and vegetables from the diary (in grams) divided by 150 and 75 respectively.

### Data management

A standard protocol for data entry was developed to ensure missing and ambiguous data were handled consistently. Ten percent of all data entered were re-checked to identify any errors. Approximately five percent of the 7-day diaries were re-collated to check for errors. The rate of agreement between the five percent of the diaries collated twice was 95.3 per cent.

### Data analysis

All data were analysed using SPSS 12.0.1 (SPSS Inc.). Statistical significance was accepted at p < 0.05. Data obtained from each of Tests 1 and 2 and the 7-day diaries were converted to 12 scores (Table 1). Scores from Test 1 were compared to those from Test 2 and to those from the diaries. Median, possible and observed score range and a target healthy score created using healthy eating guidelines [24,25], for each of the 12 scores, are shown in Table 2. The majority of scores were not normally distributed hence non-parametric tests were used in analysis for consistency.

**Table 2:** The target healthy value, possible value range, range observed, and median and interquartile range (IQR) at Test 1 for the 12 scores

**Category ***Score*	Target healthy value	Possible value range (range observed)	Test 1 Median value (IQ R)
**Intake**			
*Noncore food*	≤ 1	0–33 (0–11.9)	2.9 (2.0–4.5)
*Sweetened beverages*	≤ 1.3	0–14 (0–6)	1.6 (0.8–2.7)
*Sweetened beverages – diet soft drink*	≤ 1.3	0–11 (0–6)	1.3 (0.6–2.4)
*Water*	4	0–4 (0–4)	3 (2–4)
*Fruit*	≥ 6	1–14 (1–12)	5 (4–7)
*Vegetables*	≥ 8	1–11 (1–10)	4 (3–6)
**Healthy behaviour**			
*Healthy behaviour*	≥ 18	8–24 (8–24)	15 (12–18)
**Attitude**			
*Fruit*	≥ 16	4–20 (4–20)	18 (16–19)
*Vegetable*	≥ 16	4–20 (4–20)	16 (13–18)
**Environment**			
*Fruit & vegetable*	≥ 19	5–24 (8–24)	19 (17–21)
**Knowledge**			
*Fruit*	2	2^Φ^	4 (3–4)
*Vegetables*	3	3^Φ^	4 (4–4)

^Φ^No possible value range as score is one item only

Height and weight were converted to body mass index (BMI; kg/m^2^) and BMI was used to determine weight status (healthy, overweight or obese) using the IOTF BMI cut-points [26].

#### Reliability

Cronbach's alpha was used to assess the reliability of Test 1 scores with respect to (a) how well the individual items of the scores fit together and (b) whether they assess the same construct [18,27]. This is also referred to as internal consistency. Internal consistency is used to assess reliability of more abstract scales and as food intake is not an abstract concept this analysis was not performed on intake scores. A scale has been defined as having good internal consistency if Cronbach's alpha is above 0.7 [27]. Reliability of the scores can also be examined by determining the impact on the alpha value of removing each individual item in turn. An alpha value higher than the final value suggests the removed item is unnecessary. Items identified as unnecessary were removed from the scores and median, ranges and target healthy scores were re-calculated using the modified scores. Questionnaire questions corresponding to these unnecessary items were subsequently removed from the questionnaire.

To determine test-retest reliability at the individual level, the intra-class correlation coefficient (ICC) was used to assess absolute agreement between modified scores from Tests 1 and 2 [18]. The Wilcoxon signed rank test was used to assess test-retest reliability of modified scores at the group level.

#### Relative validity

Association between modified scores from Tests 1 and the 7-day diaries at the individual level was assessed using the Spearman correlation. For each score bias was calculated as the mean of the difference between the scores from Test 1 and the 7-day diaries, and limits of agreement as twice the standard deviation of the difference above and below this mean. A regression analysis was performed if bias was relatively large to identify if the slope of the bias line was significantly different from zero and hence not consistent. The Wilcoxon signed rank test was used to assess validity of modified scores at the group level.

## Results

One hundred and forty-one students of a potential 243 consented to the study (58%). Of these, 134 completed Test 2 and comprise the reliability study and 117 (85%) returned the 7-day diaries and comprise the validity study. Sixty-two percent of the sample was female and approximately one-third came from each grade (grade five: 36%, six: 33%, seven: 31%). Sixty-six percent attended metropolitan schools and 61% attended public schools. Fourteen percent of the sample was defined as overweight (9% boys, 17% girls) and 6 percent obese (4% boys, 8% girls).

Target healthy scores, possible score ranges and medians and ranges for modified scores observed at Test 1 are presented (Table 2). With the exception of the noncore food and vegetable intake score, the target healthy scores are similar to the median scores. For the majority of scores, observed score range at Test 1 is similar to possible range. The exceptions are the noncore food and sweetened beverage scores which were concentrated at the lower end of the range.

### Reliability

#### Internal consistency (n = 141)

Four items in three scores were identified as unnecessary. These were frequency of eating breakfast and fast food in the healthy behaviour score, attitude to cost of fruit in the fruit attitude score, and number of fruits and vegetables never consumed or not known in the fruit and vegetable environment score. After modification Cronbach alpha values were 0.50 for healthy behaviour and fruit and vegetable environment scores, 0.74 for vegetable attitude score and 0.80 for fruit attitude score.

#### Test-retest reliability (n = 134)

Test 2 occurred between eight and 36 days after Test 1 (mean = 24 days). Results for test-retest reliability are shown in Table 3. ICCs ranged from 0.47 to 0.66 for 10/12 scores (p < 0.001). The fruit and vegetable knowledge scores had ICCs less than 0.4 (p < 0.05 and p < 0.001 respectively). The Wilcoxon signed rank test found no evidence of a difference between means (p > 0.05) for 10 out of 12 scores at Tests 1 and 2. There was evidence of a difference between mean sweetened beverage and water intake scores (p < 0.001) (Table 3).

**Table 3:** Test-retest reliability and mean values for Test 1 and Test 2 for the 12 scores

Score	Intraclass correlation	95% confidence interval	Mean scores	
			
			Test 1	Test 2
**Intake**				
Noncore food	0.47**	0.31–0.60	3.5	3.1
Sweetened beverages	0.59**	0.46–0.70	1.8	1.5*
Sweetened beverages – diet soft drink	0.63**	0.50–0.72	1.6	1.4
Water	0.57**	0.44–0.68	3.0	3.3**
Fruit	0.66**	0.55–0.75	5.4	5.3
Vegetables	0.66**	0.55–0.75	4.5	4.3
**Healthy behaviour**				
Healthy behaviour	0.64**	0.51–0.75	15.1	14.9
**Attitude**				
Fruit	0.50**	0.36–0.62	21.1	20.9
Vegetable	0.62**	0.50–0.72	15.1	15.1
**Environment**				
Fruit & vegetable	0.59**	0.45–0.69	18.9	18.3
**Knowledge**				
Fruit	0.16*	-0.01–0.32	3.8	3.6
Vegetables	0.36**	0.20–0.49	4.0	3.8

*Correlation/difference in means significant at the 0.05 level

**Correlation/difference in means significant at the 0.001 level

### Relative validity (n = 117)

Results for relative validity are shown in Table 4. Spearman correlations ranged from 0.34 to 0.48 (p < 0.01). Mean bias ranged from -1.2 to 0.6. The scores with the greatest bias were sweetened beverages, sweetened beverages minus diet drinks and fruit intake (-1.2, -1.1 and 1.0 respectively). For three scores (noncore foods and sweetened beverages with and without diet drinks) regression analysis indicated proportional bias. That is, as consumption of sweetened beverages (with and without diet drinks) increased, the bias decreased, while as consumption of noncore foods increased, the bias increased. The greatest difference between the upper and lower limits of agreement was observed for the sweetened beverage and healthy behaviour scores. There was evidence of a difference between means for four of seven scores from Test 1 and the 7-day diaries, as assessed by the Wilcoxon signed rank test (Table 4).

**Table 4:** Relative validity and mean values for Test 1 and the 7-day diaries for the 7 scores

Score	Spearman correlation**	Bias (Test 1-diaries)	Limits of agreement	Mean values	
				
				Test 1	7-day diary
**Intake**					
Noncore food	0.36	0.6	-4.3, 5.5	3.4	2.7*
Sweetened beverages	0.34	-1.2	-7.0, 4.6	1.8	2.9***
Sweetened beverages – diet soft drink	0.38	-1.1	-6.4, 4.2	2.9	2.6***
Water	0.42	-0.1	-2.2, 2.0	2.9	3.1
Fruit	0.48	1.0	-3.5, 5.1	5.3	4.2***
Vegetables	0.36	0.5	-3.4, 4.4	4.5	4.1
**Healthy behaviour**					
Healthy behaviour	0.46	-0.4	-6.3, 5.5	15.0	15.5

* Mean difference significant at the 0.05 level

** All significant at the 0.01 level

*** Mean difference significant at the 0.001 level

## Discussion

The purpose of this study was to determine the reliability and relative validity of a new child nutrition questionnaire which simultaneously measures dietary patterns known to increase the risk of positive energy balance, and food behaviours, attitudes, environments and knowledge in school children aged 10–12 years. This study is important because of the lack of current, published valid and reliable tools that measure all of these factors and have been assessed psychometrically.

After modification, all scores analysed were found to have reasonable to good internal consistency. Test retest-reliability was good for all scores except fruit and vegetable knowledge. Relative validity was lower but still determined as acceptable for all scores, based on comparisons to results in similar studies.

Only the fruit and vegetable attitudes scores had alpha values above 0.7, the recommended value for good internal consistency [27]. Results from previous studies are variable. The Family Eating and Activity Habits Questionnaire, measuring food behaviours and environments, found consistently higher alpha values with alpha above 0.7 for all subscales (n = 40 mothers of children 6–11 years, Israel) [28]. Three similar questionnaires had ranges similar to the present study with slightly greater maximums. One questionnaire measuring family and peer influences on fruit, juice and vegetable consumption [29] (n = 210 Year 4–6 students, Texas) had a range of 0.42–0.89 while another measuring food attitudes and environments [30] (n = 328 10–11 year olds, five European countries) reported a range of 0.45–0.92. Finally, a questionnaire measuring theoretical constructs believed to predict fruit and vegetable consumption amongst sixth graders (n = 129 sixth graders, Norway) had alpha ranging from 0.41 to 0.81 [31]. The reason for some very high values in previous studies is unclear. Cronbach alpha values are quite sensitive to the number of items in a scale and values lower than 0.7 are common with less than 10 items [27]. The five scores assessed for internal consistency in our study had less than 10 items which could explain why the majority of alpha values were less than 0.7. Removal of unnecessary score items not only improved internal consistency of these scores, but also meant the corresponding questions could be removed from the questionnaire, [see Additional file 1] hence reducing subject and researcher burden.

Nine out of 12 scores in this study had good test-retest reliability with an ICC > 0.5 (range: 0.50–0.66) (p < 0.001). In general, this is equivalent to test-retest reliability of similar questionnaires. One identified constructs believed to predict fruit and vegetable consumption among sixth graders [31] and included items regarding fruit and vegetable attitudes, preferences, behaviours, self-efficacy and intention to eat. However, unlike the present questionnaire, it did not measure actual fruit and vegetable intake. Spearman correlations were comparable to our study, ranging from 0.51 to 0.79 (p-values not reported). The higher maximum correlation could be due to the shorter time interval between Test 1 and Test 2 (14 days compared with 24 days in our study) implying students were more able to remember their answers.

Similar to our study, other studies have reported poor to good results for test-retest reliability indicating considerable variability in children of this age group. A self-report instrument used in children in school years four to six in a similar setting (Australian primary school, n = 245) reported kappa statistics ranging from 0.18–0.63 for individual questionnaire items regarding food intake and purchasing practices at school [32]. Another study reported Pearson correlations less than 0.5 for five of eight subscales [29], while another, which also had a shorter time interval between tests than our study (7–12 days) found ICCs greater than 0.5 for all constructs [30]. In contrast, The Family Eating and Activity Habits questionnaire reported higher test-retest reliability than our study, ranging from 0.78–0.90 (p < 0.01) however this was a parent completed questionnaire [28].

The fruit and vegetable knowledge scores in the present study had poor test-retest reliability with ICCs < 0.4 (p < 0.05) and consequently the questions used to create these scores have been removed from the questionnaire [see Additional file 1]. While Spearman correlations of 0.40–0.56 were reported for the food knowledge questions in a School-based Nutrition Monitoring Student Questionnaire (n = 254 Year 8 students, Texas) [21] these items had the lowest test-retest reliability correlations in this questionnaire (except for four attitude questions), suggesting that when compared to other constructs, knowledge may be less reliable to measure in school children.

Relative validity was not as strong as reliability. However, three scores did demonstrate validity at the group level. It is our position that validity at the group level is of more relevance as data collected using this tool in the community-based intervention will be analysed at the group level. Additionally, our results for relative validity are comparable to similar studies, i.e. this tool is as good as other similar tools in terms of its validity – a rationale used previously [33].

One study was identified that assessed the validity of a self-reported measure of fruit and vegetable intake among sixth graders [33] and Spearman correlations ranged from 0.21 to 0.32 (p < 0.001). Validity was better in our study with Spearman correlations ranging from 0.34 to 0.48. The food diary used to validate this tool [33] asked about consumption of 277 food items, even though the questionnaire was solely about fruit and vegetable intake. This is in contrast to our food diary which only asked about foods in the questionnaire. The school-based Nutrition Monitoring Student Questionnaire (n = 209 Year 8 students, Texas) was developed through a process including a needs assessment and focus group testing [21]. It addressed nutrition attitudes and behaviours (dietary fat intake, consumption of fruit and vegetables, grain products and high calorie/low nutrient foods) [21]. Spearman correlations ranged from 0.32–0.68 with only five of 17 correlations being less than 0.5 [21]. The majority of these correlations are higher than those in our study; this could reflect the older age of the children in this study, as other studies have shown the accuracy of dietary intake reporting to increase with age [34].

The Youth Healthy Eating Index (n = 12 452 9–14 year olds, across the United States of America) was derived from a 132 item food frequency questionnaire and it focuses on both food behaviours and intake of 'healthful' and 'unhealthful' foods [35]. It has similar validity to the present study with a Pearson correlation of 0.44 [35]. While this questionnaire is different to that used in our study, some aspects of the tools are comparable, for example the use of school children in the school setting, and that both provide a broad picture of dietary intake by looking at more than one food group. Similarly, a school-food checklist measuring food intake (SFC) at school (n = 910 school children aged 9–12 years, Australia) was validated using a weighed food record (WR) [36]. The Spearman correlation for total energy intake compared between the SFC and the WR was 0.77 (p < 0.01) [36], much higher than the validity correlations in our study, possibly reflecting that this study looked at intake over a much shorter time period (1 day) compared to our study (1 week).

Mean bias indicates that compared with the 7-day diaries, the questionnaire overestimates fruit, vegetable and noncore food intake and underestimates sweetened beverage intake with and without diet drinks. These results for fruit and vegetable intake are consistent with another study that found the test method (parent report of child intake) to overestimate intake of core foods, including fruit and vegetables, compared with the reference method (food diary)(n = 50 parents of children 5–10 years, Australia) [37]. Children have difficulty estimating portion size [38,39] and may conceptualise one instance of consumption to represent one serve, which is not always the case. Hence overestimation of fruit and vegetable intake by the questionnaire is predictable and does not represent a flaw in the questionnaire itself or the methodology used.

As the regression analysis revealed no significant difference between the slope of the bias line and zero for fruit and vegetable intake, this bias is constant and hence can be easily adjusted for. In contrast there was a proportional bias for three scores, noncore food and sweetened beverages with and without diet drinks, and this cannot be easily adjusted for. However, the size of the bias must be considered in the context of the scores, e.g. the noncore foods score bias is low enough in the context of the large score range to be of little practical significance.

Limits of agreement were reported because this is the recognised correct method of analysis for agreement studies [40]. However, absolute limits are only of value if applying and analysing the data from the tool at the individual level [40]. This is not the purpose of this tool and hence the results are less relevant.

This study has a number of strengths. The large sample size, which is comparable to [21,29,31,33] or more than [28] previous studies. The high diary return rate means that data used for validation are likely to be representative of the larger sample. The food diary used to validate the questionnaire was designed by the authors to reflect the questionnaire content but was not a validated tool itself. However, a food diary is an accepted standard for validation [41] and validation requires that a reference method be more accurate then a test method [41]. The food diary covered a week (the period of interest for many questionnaire items). As it was completed at the time of consumption it did not rely on memory and thus was a more accurate assessment of behaviour and hence appropriate for validation. Furthermore, as this food diary was designed specifically to reflect the questionnaire, it has the ability to demonstrate that the questionnaire is a valid representation of intake. Estimating actual fruit and vegetable intake using the NNS database for 9–13 year olds was a resourceful use of data collected and avoided increased subject burden. The standard protocol for data entry and data checking ensured a low error rate with respect to the questionnaires. Finally, the use of internal consistency adds to the more traditional test-retest reliability analysis.

There are nevertheless several limitations to the study. The complexity of the questionnaire posed a challenge when designing the diary with the result that the diary was not sensitive to all questions in the questionnaire. For example, as a 7-day diary it could not distinguish between frequencies of less than once per week and never/rarely. Hence these two questionnaire categories had to be combined for data analysis and validity and reliability has only been demonstrated with combined categories. These categories have been combined on the questionnaire for future use. The diaries only identified whether a subject consumed canned or dried fruit, not the type of canned or dried fruit as identified in the questionnaire. Consequently, canned or dried fruit was given one point for variety in the diaries, while in the questionnaire the total number of different fruit, regardless of type, was summed. Hence canned or dried fruits with more than one fruit type would have been given a higher variety score by the questionnaire. While potentially the total fruit variety scores are lower from the diaries, this is unlikely to have an overall effect because scores were quantiled and a small change in score would not lead to a change in category.

While not a limitation, the complexity of the 7-day diary, reflecting the complexity of the questionnaire, must be acknowledged. This made diary collation difficult and inevitably errors were identified when the five percent were re-collated. Converting continuous data from the diaries into categorical data to reflect the questionnaire was challenging and inevitably accuracy was compromised. While some students had difficulty completing the questionnaires, help was available during Tests 1 and 2 so this is unlikely to have compromised test-retest reliability. However, the increased complexity of the diary and lack of trained helpers when it was filled out at home needs to be acknowledged as a potential source of error. This is supported by the raw data with inconsistencies in completion of diary questions from day to day. Hence, relative validity is potentially higher than the results suggest.

The modified child nutrition questionnaire for measurement of dietary patterns associated with the risk of positive energy balance and food behaviours, attitudes and environments is a valid and reliable tool to assess these parameters in a population of school children aged 10–12 years participating in the community-based obesity prevention project. Hence the data collected using this tool during this project will contribute to the evidence regarding effectiveness of healthy eating interventions integral to prevention of overweight and obesity.

This conclusion is justified by the good results for reliability and the identified complexities in the measurement of validity. Additionally, the preceding comparisons between this study and others highlight that there is a lack of similar tools in the published literature that measure the range of factors of our tool. Furthermore, the majority of similar studies are predominantly single setting (i.e. the school or home) while this study considers multiple environments. Finally, not only are the number of similar tools in the published literature limited, but even fewer have been assessed psychometrically. Hence there is no standard to which the reliability and validity results of this questionnaire can be compared and hence the results of this study set a new standard for similar studies in the future.

This questionnaire was designed specifically for evaluation of this community-based childhood obesity prevention project and is based on the key messages and strategies of this intervention (Figure 1). While these key messages and strategies are likely to be relevant to other childhood obesity interventions, they are not necessarily directly transferable. Thus there is an urgent need to (a) develop similar tools that measure multiple parameters of interest to professionals in childhood obesity prevention including diet dietary patterns that increase the risk of positive energy balance, and food attitudes, behaviours, environments and knowledge associated with healthy eating and (b) for such tools to be assessed psychometrically. A key challenge in this area is assessment of validity of parameters that do not lend themselves to traditional validation such as behaviours, attitudes and knowledge.

## Conclusion

The child nutrition questionnaire reported in this study is a valid and reliable tool in a sample of school children aged 10–12 years involved in this community-based obesity prevention project. It is unique because it (a) simultaneously assesses dietary patterns known to increase the risk of positive energy balance, and food behaviours, attitudes, environments and knowledge associated with healthy eating in school children, (b) assesses these factors across multiple sites including the home and school and (c) has been assessed for reliability and relative validity and had psychometric properties reported. This study contributes new knowledge to the field of diet methodology and sets a new standard for reliability and relative validity of similar tools, particularly those used in comparable community-based obesity prevention interventions. The findings have allowed the development of a better tool, which obtains useful data in a less burdensome way.

## Competing interests

The authors declare that they have no competing interests.

## Authors' contributions

All authors read and approved the final manuscript. AM and NM developed the questionnaire, AW and NM recruited subjects, AW assisted with data entry and collated the 7-day diaries, AM and AW developed the 7-day diaries and scores and AW undertook the data analysis. AW wrote the first draft of the manuscript and it was revised by all three authors.

## Supplementary Material

Additional file 1 Modified Child Nutrition Questionnaire. The Child Nutrition Questionnaire after removal of questionnaire items found to be unnecessary (as defined by Cronbach alpha values) or with poor test-retest reliability. Click here for file
