# Supplementary material for: Reliability and relative validity of a child nutrition questionnaire to simultaneously assess dietary patterns associated with positive energy balance and food behaviours, attitudes, knowledge and environments associated with healthy eating
Source: Int J Behav Nutr Phys Act. 2008 Jan 29;5:5. doi: 10.1186/1479-5868-5-5 (PMC2268941; doi:10.1186/1479-5868-5-5)
Supplement: Additional file 1 — Modified Child Nutrition Questionnaire. The Child Nutrition Questionnaire after removal of questionnaire items found to be unnecessary (as defined by Cronbach alpha values) or with poor test-retest reliability. [file 1479-5868-5-5-S1.doc]

**Modified child nutrition questionnaire**

**
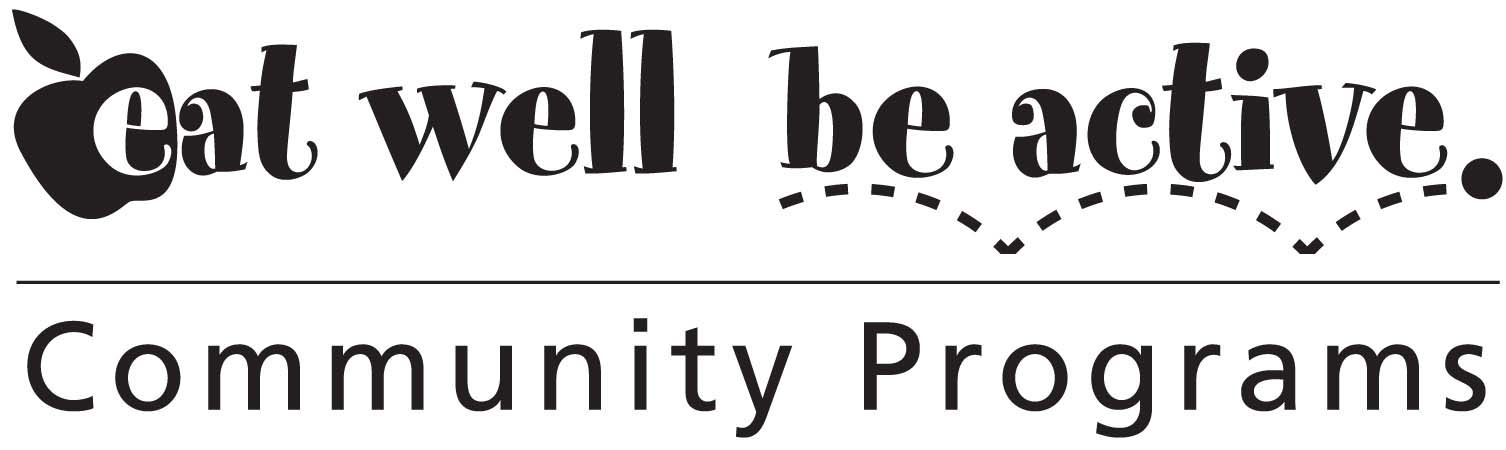
**

**Nutrition Questionnaire for students in Years 5, 6, 7**

This questionnaire asks about what you eat and drink. It starts with some general questions about what you think about fruit and vegetables and then about what you eat and drink at different times.

The teacher and classroom helpers will help you fill out this questionnaire.

The information will be used to describe all of the children in years 5, 6 & 7 as a group. Your individual information will be kept private and confidential.

**Your Name___________________________________________________________________________**

***Reference Number*** 🞎🞎🞎🞎🞎🞎-🞎

**Year level (for example yr 6) _________________________________________________________**

What you usually eat and drink

1. Think about today. Describe what you drink at each time?

# Tick as many boxes as apply

| **Drinks you will have/have had today at:** | **Recess**  1 | **Lunch**  2 | **After school *(not including dinner)***  3 |
| --- | --- | --- | --- |
| 1. Nothing to drink |  |  |  |
| 1. Cordial |  |  |  |
| 1. Fruit Juice or fruit juice drink |  |  |  |
| 1. Regular Soft Drink or Energy/Sports   Drink (eg. *Powerade*) |  |  |  |
| 1. Diet Soft Drink (eg Coke Zero, Diet Fanta) |  |  |  |
| 1. Water |  |  |  |
| 1. Milk/flavoured milk |  |  |  |

1. Think about today. Describe what you eat at each time?

# Tick as many boxes as apply

| **Foods you will have/have eaten today at:** | **Recess**  1 | **Lunch**  2 | **After school *(not including dinner)***  3 |
| --- | --- | --- | --- |
| 1. Nothing to eat |  |  |  |
| 1. Potato crisps or similar snack (eg twisties) |  |  |  |
| 1. Chocolate/Chocolate bar |  |  |  |
| 1. Lollies |  |  |  |
| 1. Muesli bar/ fruit bar |  |  |  |
| 1. Yoghurt / custard |  |  |  |
| 1. Savoury biscuits (eg saladas, jatz & dip) |  |  |  |
| 1. Sweet biscuits/Cake/Muffin/Doughnut |  |  |  |
| 1. Icecream/Iceblock |  |  |  |
| 1. Vegetables or salad |  |  |  |
| 1. Fruit (fresh or canned) |  |  |  |
| 1. Dried fruit (eg sultanas) |  |  |  |
| 1. Hot chips/French fries/wedges |  |  |  |
| 1. Pie/Pastie/Sausage roll |  |  |  |
| 1. Hot dog |  |  |  |
| 1. Pizza |  |  |  |
| 1. Sandwich/roll |  |  |  |
| 1. Bread/toast |  |  |  |
| 1. Spaghetti/pasta/noodles/rice |  |  |  |
| 1. Soup |  |  |  |

1. In the class room, how often do you have a ‘fruit and/or vegetable break’?

# *Tick one box*

# Never/rarely Once/week Most days/week Every day

1 2 3 4

1. In class time, do you usually have a drink on your desk?

# *Tick one box*

1.  Not allowed

2.  No, even though it is allowed

3.  Yes - If yes, what do you usually drink? ________________

1. How often do you usually do the following?

## Tick one box in each row

|  | **Never/rarely OR**  **Less than once/week**  1 | **About 1-3 times/week**  2 | **About 4-6 times/week**  3 | **Every day**  4 |
| --- | --- | --- | --- | --- |
| a. Drink water |  |  |  |  |
| b. Drink fruit juice or fruit juice drink |  |  |  |  |
| c. Drink softdrink (not including *diet* softdrink) |  |  |  |  |
| d. Carry a water bottle |  |  |  |  |
| e. Eat chocolate or lollies |  |  |  |  |
| f. Eat hot chips/French fries/wedges |  |  |  |  |
| g. Eat potato crisps or similar snack (eg twisties) |  |  |  |  |
| i. Help choose or buy groceries for the family |  |  |  |  |
| j. Help prepare your dinner |  |  |  |  |
| k. Eat dinner with most of the family |  |  |  |  |
| l. Eat dinner in front of the television |  |  |  |  |
| m. Eat snacks in front of the television |  |  |  |  |

What do you think about

1. How strongly do you agree or disagree with the following statements?

*Tick one box in each row*

|  | Strongly agree  1 | **Agree**  2 | **Not sure**  3 | **Agree**  4 | **Strongly disagree**  *5* |
| --- | --- | --- | --- | --- | --- |
| a. Eating vegetables makes me feel healthy |  |  |  |  |  |
| b. I like the taste of many vegetables |  |  |  |  |  |
| c. In my home, vegetables are served at dinner most nights |  |  |  |  |  |
| d. I like tasting new vegetables that I haven’t tried before |  |  |  |  |  |
| e. It is easy to prepare vegetables to eat eg make a salad |  |  |  |  |  |
| f. Eating fruit makes me feel healthy |  |  |  |  |  |
| g. I like the taste of most fruit |  |  |  |  |  |
| h. Fruit is an easy snack |  |  |  |  |  |
| i. I like tasting new fruits that I haven’t tried before |  |  |  |  |  |
| j. In my home fruit is available to eat at any time |  |  |  |  |  |
| l. I like to drink water |  |  |  |  |  |
| m. I ask my parents to buy foods or drinks that I see advertised on television |  |  |  |  |  |
| n. My parents encourage me to eat fruit and vegetables |  |  |  |  |  |
| o. Most of my teachers encourage the students to eat fruit and vegetables |  |  |  |  |  |

1. Where did you/will you get your recess from today?

# *Tick one box*

**Home Canteen Shop outside of school Friends No recess today**

1 2 3 4 5

1. Where did you/will you get your lunch from today?

# *Tick one box*

**Home Canteen Shop outside of school Friends No lunch today**

1 2 3 4 5

About fruit and vegetables you eat

1. How many serves of vegetables do you usually eat each day?

(1 serve = 1 cup of salad vegetables, OR ½ a cup of cooked vegetables, OR 1 medium potato)

# *Tick one box*

1. I don’t eat vegetables
2. Less than 1 serve a day
3. 1-2 serves a day
4. 3-5 serves a day
5. More than 5 serves a day
6. How many serves of fruit do you usually eat each day?

(1 serve = 1 medium piece, OR 2 small pieces of fruit eg mandarins or apricots, OR 1 cup of diced pieces)

# *Tick one box*

1. I don’t eat vegetables
2. Less than 1 serve a day
3. 1-2 serves a day
4. 3-5 serves a day
5. More than 5 serves a day
6. Below is a list of different types of fruits (fresh, canned or dried). For each fruit please indicate answer PART A and PART B.

**PART A: Please indicate if you ate this fruit yesterday, by ticking the box that applies to you, for each fruit**

**PART B: Please indicate if you like this fruit by ticking the box that applies to you, for each fruit**

## Tick one box in each row

|  | **PART A** | | **PART B** | | |
| --- | --- | --- | --- | --- | --- |
| **Type of fruit** | **I ate this fruit yesterday**  1 | **I didn’t eat this fruit yesterday**  2 | **Never had it or don’t know what it is**  3 | **Yes I like this fruit**  4 | **No I don’t like this fruit**  5 |
| 1. Apple |  |  |  |  |  |
| 1. Apricot |  |  |  |  |  |
| 1. Banana |  |  |  |  |  |
| 1. Grapes |  |  |  |  |  |
| 1. Kiwi fruit |  |  |  |  |  |
| 1. Mandarin |  |  |  |  |  |
| 1. Nectarine |  |  |  |  |  |
| 1. Orange |  |  |  |  |  |
| 1. Peach |  |  |  |  |  |
| 1. Pear |  |  |  |  |  |
| 1. Pineapple |  |  |  |  |  |
| 1. Plum |  |  |  |  |  |
| 1. Rockmelon |  |  |  |  |  |
| 1. Strawberries |  |  |  |  |  |
| 1. Watermelon |  |  |  |  |  |

1. Below is a list of different types of vegetables (fresh, canned or frozen).
   For each vegetable please answer PART A and PART B

**PART A: Please indicate if you ate this vegetable yesterday, by ticking the box that applies to you, for each vegetable**

**PART B: Please indicate if you like this vegetable by ticking the box that applies to you, for each vegetable**

# Tick one box in each row

| **Type of vegetable** | **I ate this vegetable yesterday**  1 | **I didn’t eat this vegetable yesterday**  2 |
| --- | --- | --- |
| a. Beans (green) |  |  |
| b. Beetroot |  |  |
| c. Broccoli |  |  |
| d. Brussel sprouts |  |  |
| e. Cabbage |  |  |
| f. Capsicum |  |  |
| g. Carrot |  |  |
| h. Cauliflower |  |  |
| i. Celery |  |  |
| j. Chinese greens |  |  |
| k. Corn |  |  |
| l. Cucumber |  |  |
| m. Eggplant |  |  |
| n. Legumes (baked beans, chickpeas, lentils, kidney beans) |  |  |
| o. Lettuce |  |  |
| p. Mushroom |  |  |
| q. Peas |  |  |
| r. Pot ato (not hot chips) |  |  |
| s. Potato fried eg hot chips/ french fries/wedges |  |  |
| t. Pumpkin |  |  |
| u. Spinach |  |  |
| v. Sweet potato |  |  |
| w. Tomato |  |  |
| x. Zucchini |  |  |
| y. Squash |  |  |

**☺ Fantastic, you’ve finished**

**THANK YOU**
